# RETRACTION: Silencing Circular RNA VANGL1 Inhibits Progression of Bladder Cancer by Regulating miR‐1184/IGFBP2 Axis

**DOI:** 10.1002/cam4.71178

**Published:** 2025-08-25

## Abstract

RETRACTION: YangD. , QianH. , FangZ. , XuA. , ZhaoS. , LiuB. and LiD. , “Silencing Circular RNA VANGL1 Inhibits Progression of Bladder Cancer by Regulating miR‐1184/IGFBP2 Axis,” Cancer Medicine 9, no. 2 (2020): 700–710, 10.1002/cam4.2650.31758655 PMC6970048

The above article, published online on 23 November 2019 in Wiley Online Library (wileyonlinelibrary.com), has been retracted by agreement between the journal Editor‐in‐Chief, Stephen Tait; and John Wiley & Sons Ltd. The retraction has been agreed upon due to image overlaps within Figure 2F, as well as between images presented in Figures 2E and 5E. Furthermore, an image from Figure 5F appeared in another article, depicting a different scientific context, and the GAPDH western blot bands in Figure 2B were also identified in a separate publication. Both duplications were found in earlier articles published by different author groups. While the authors cooperated with our investigation and provided some data, they were unable to explain the duplications found in other articles. Due to the nature and extent of these concerns, the editors have lost confidence in the results and conclusions reported in this study. The authors disagree with the retraction.

RETRACTION: D. Yang , H. Qian , Z. Fang , A. Xu , S. Zhao , B. Liu and D. Li , “Silencing Circular RNA VANGL1 Inhibits Progression of Bladder Cancer by Regulating miR‐1184/IGFBP2 Axis,” Cancer Medicine 9, no. 2 (2020): 700–710, 10.1002/cam4.2650.31758655 PMC6970048

The above article, published online on 23 November 2019 in Wiley Online Library (wileyonlinelibrary.com), has been retracted by agreement between the journal Editor‐in‐Chief, Stephen Tait; and John Wiley & Sons Ltd. The retraction has been agreed upon due to image overlaps within Figure 2F, as well as between images presented in Figures 2E and 5E. Furthermore, an image from Figure 5F appeared in another article, depicting a different scientific context, and the GAPDH western blot bands in Figure 2B were also identified in a separate publication. Both duplications were found in earlier articles published by different author groups. While the authors cooperated with our investigation and provided some data, they were unable to explain the duplications found in other articles. Due to the nature and extent of these concerns, the editors have lost confidence in the results and conclusions reported in this study. The authors disagree with the retraction.

